# Consensus Guideline for the Diagnosis and Treatment of Tyrosine Hydroxylase (TH) Deficiency

**DOI:** 10.1002/jimd.70106

**Published:** 2025-11-10

**Authors:** Mariya Sigatullina Bondarenko, Oya Kuseyri Hübschmann, Jan Kulhánek, Roser Pons, Toni S. Pearson, Kathrin Jeltsch, Ivana Badnjarevic, Tessa Wassenberg, Gabriella Horvath, Galina Stevanovic, Manju A. Kurian, Elisenda Cortès‐Saladelafont, Agathe Roubertie, Vincenzo Leuzzi, Mariarita Bertoldi, Mario Mastrangelo, Birgit Assmann, THD Guidelines Working Group, Ines Denzler, Clarisa Maxit, Natalia Alexandra Julia‐Palacios, Shekeeb Mohammad, Helly Goez, Wai‐Ian Yeung, Tomáš Honzík, Ivana Kavecan, Alba Tristán‐Noguero, Alberto Burlina, Daniela Gueraldi, Sushil Bandodkar, Willemsen Michel, Francesco Porta, Sheila Wong, Filippo Manti, Reem Alkhater, Chris Mühlhausen, Alice Kuster, Dimitrios Zafeiriou, Paulo Sgobbi, Wladimir Bocca Vieira de Rezende Pinto, Angeles Garcia‐Cazorla, Thomas Opladen

**Affiliations:** ^1^ Neurometabolic Unit and Synaptic Metabolism Lab, Neurology Department Institut Pediàtric de Recerca, Hospital Sant Joan de Déu Barcelona Spain; ^2^ Neuropediatric Unit, Pediatric Department Hospital Universitari Germans Trias I Pujol Badalona Spain; ^3^ Heidelberg University, Medical Faculty Heidelberg, Center for Paediatric and Adolescent Medicine Department I, Division of Pediatric Neurology and Metabolic Medicine Heidelberg Germany; ^4^ Department of Pediatrics and Inherited Metabolic Disorders Charles University and General University Hospital in Prague Prague Czech Republic; ^5^ First Department of Paediatrics Agia Sofia Hospital, National and Kapodistrian University of Athens Athens Greece; ^6^ Division of Neurology, Nationwide Children's Hospital The Ohio State University College of Medicine Columbus Ohio USA; ^7^ Lil' Brave One (Hrabrisa)‐Patient Organization for Rare Neurotransmitter Diseases Novi Sad Serbia; ^8^ Department of Pediatrics, Pediatric Neurology and Metabolic Unit, Research Group Vitality Universitair Ziekenhuis Brussel Brussels Belgium; ^9^ Division of Biochemical Genetics Vancouver General Hospital & BC Children's Hospital Vancouver British Columbia Canada; ^10^ Clinic for Neurology and Psychiatry for Children and Youth Belgrade Serbia; ^11^ Developmental Neurosciences, Zayed Centre for Research, UCL GOS‐ICH; Department of Neurology Great Ormond Street Hospital London UK; ^12^ Pediatric Department Son Llàtzer University Hospital Palma (Mallorca) Spain; ^13^ Neuropediatric Service CHU Montpellier Montpellier France; ^14^ Department of Human Neuroscience, Child Neurology and Psychiatry Sapienza University Rome Italy; ^15^ Department of Neuroscience, Biomedicine and Movement Sciences Verona University Verona Italy; ^16^ Child Neurology and Psychiatry Unit, Department of Neuroscience/Mental Health Policlinico Umberto I Rome Italy

**Keywords:** dopa‐responsive dystonia, international working group on neurotransmitter related disorders, neurotransmitter, tyrosine hydroxylase deficiency

## Abstract

Tyrosine hydroxylase (TH) catalyses the rate‐limiting step in dopamine biosynthesis. Autosomal recessive tyrosine hydroxylase deficiency (THD) leads to clinical phenotypes reflecting the deficiency of dopamine, norepinephrine, or epinephrine in the central nervous system (CNS), presenting along a continuous spectrum from mild to severe forms of the disease. The diagnosis is suggested by the detection of low CSF homovanillic acid (HVA) and confirmed by identifying biallelic pathogenic variants in the TH gene. L‐dopa/decarboxylase inhibitor (DCI) supplementation is often the first‐line treatment, and most patients have a good therapeutic response. However, initiation of therapy can be challenging in patients with severe disease forms who develop L‐dopa/DCI‐induced dyskinesia. Therefore, alternative treatment options, such as monoamine oxidase (MAO) inhibitors, must be evaluated to optimize motor symptom control. Clinical experience suggests that early diagnosis and treatment initiation may improve the outcome. Additionally, a multidisciplinary treatment approach should be utilized to monitor neurocognitive development and other comorbidities that may occur in THD. In this consensus guideline, representatives of the International Working Group on Neurotransmitter related Disorders (iNTD) and patient advocates evaluated all the evidence available in the literature on the diagnosis and management of THD and developed recommendations using the SIGN and GRADE methodologies. Based on the limited evidence, practical recommendations have been developed to support clinical diagnosis, laboratory testing, neuroimaging, medical treatment, and non‐medical interventions. Research topics for further development were identified. This guideline aims to improve the care of patients with THD worldwide and raise general awareness of this rare disease.

**Abbreviations**
3‐OMD: 3‐O‐methyldopa
5‐HIAA: 5‐hydroxyindoleacetic acid
5‐HTP: 5‐hydroxytryptophan
5‐MTHF: 5‐methyltetrahydrofolate
AADC: aromatic l‐amino acid decarboxylase
BH_4_
: tetrahydrobiopterin
BW: body weight
CNS: central nervous system
COMT: catechol‐o‐methyl transferase
CSF: cerebrospinal fluid
DA: dopamine agonist
DBS: dry blood spot
DC: decarboxylase
DCI: decarboxylase inhibitor
EEG: electroencephalography
GPP: good practice point
GRADE: grading of recommendations, assessment, development and evaluation
HVA: homovanillic acid
iNTD: international working group on neurotransmitter related disorders
L‐dopa: l‐3,4‐dihydroxyphenylalanine
MAO(−I): monoamine oxidase (inhibitor)
MHPG: 3‐methoxy‐4‐hydroxyphenylglycol
NBS: newborn screening
NGS: next‐generation sequencing
SAM: s‐adenosyl‐methionine
SIGN: Scottish intercollegiate guideline network
SSRI: selective serotonin reuptake inhibitors
TH: tyrosine hydroxylase
TPH: tryptophan hydroxylase
Tyr: tyrosine
VLA: vanillactic acid

## 1 Background

Tyrosine hydroxylase deficiency (THD) is an ultra‐rare autosomal recessive inherited metabolic disorder caused by a deficient cerebral tyrosine hydroxylase enzyme (TH, OMIM 191290). TH is required to catalyse the conversion of tyrosine to L‐Dopa, which is the enzymatic rate‐limiting step in catecholamine biosynthesis. The most prominent clinical features can be attributed to central dopamine deficiency and include dystonia, axial hypotonia, bradykinesia, and oculogyric crises. Patients typically present in infancy with developmental delay, but milder cases may present later in childhood with symptoms like dopa‐responsive dystonia (Segawa syndrome). Most patients respond to treatment with levodopa, but the extent of treatment response is variable. A small proportion of patients do not benefit from treatment. The reason for this is not well understood, but it is not solely due to genotype, as patients with the same genotype may show a different response to therapy. Though the exact prevalence is not known, to date 190 cases of THD have been described worldwide [1, 2, 3]. According to Orphanet reports, the prevalence is 1–9/1 000 000. A total of 63 patients are collected in the patient registry of the International Working Group on Neurotransmitter‐related Disorders (iNTD, https://www.intd‐registry.org/).

Historically, THD diagnosis was based on the analysis of cerebrospinal fluid (CSF), showing depletion of dopaminergic metabolites: homovanillic acid, HVA, and 3‐methoxy‐4‐hydroxyphenylglycol (MHPG), and the genetic confirmation by molecular studies of the TH gene. However, as genetic testing techniques, such as next‐generation sequencing (NGS), clinical exome sequencing (CES), and whole exome sequencing (WES), have become more prevalent, they increasingly play a crucial role in detecting and confirming the diagnosis of THD. More than 60 disease‐causing pathogenic variants in the human TH gene have been identified [2, 3], of which the most frequent are p.Arg233His and p.Leu236Pro [3]. There is no clear correlation between the genotype and phenotype since the same variants, occurring in homozygosity or compound heterozygosity, can be associated with mild to severe phenotypes [3, 4].

Treatment with L‐dopa in combination with a peripheral decarboxylase inhibitor (DCI, e.g., carbidopa or benserazide) represents the first‐choice strategy for the treatment of patients with THD. However, treatment responses and occurrence of L‐dopa induced dyskinesia may strongly vary between individual patients [5].

In 2013, iNTD was founded with the aim of improving care for patients with neurotransmitter‐related disorders, including THD. Today (July 2025) iNTD is a growing worldwide network of 63 neurometabolic centres from 29 countries. In addition to maintaining a patient registry, one of its goals is to develop consensus guidelines for neurotransmitter‐related disorders, bringing all published evidence and experience of leading expert centres together. This guideline on diagnosis and treatment of THD is developed under the umbrella of the iNTD network. It is based on a systematic review of existing literature and aims to harmonize the care of patients with THD.

## 2 Methods

### 2.1 Composition of the Guideline Working Group and Timeline

An executive committee, including MS, AG, TO, and KJ (secretary), was formed to oversee guideline development and coordinate the subgroups on various topics. The guideline working group included 28 child neurologists, 1 child psychiatrist, 3 geneticists, 7 biochemists, and 1 research project manager (KJ). All members of the group are associated with iNTD and possess experience in diagnosing and treating THD. Additionally, a representative from a patient organization focused on rare neurotransmitter diseases, including THD (IB), took part in the guideline development.

A preliminary guideline working group meeting took place online in February 2023. A start‐up meeting took place in June 2023, Prague, Czech Republic (during the EPNS Congress). Additional in‐person meetings were held in February 2024 in Barcelona (executive committee only); in September 2024 in Porto (during SSIEM congress); in December 2024 in Belgrade (THD Guideline Meeting); and in February 2025 in Barcelona (during the Paediatric Movement Disorders Meeting).

Three lay persons read and commented on the guideline before submission. In accordance with SIGN guidelines, an external academic reviewer with expertise in neurometabolic and movement disorders was consulted to provide feedback on the draft prior to submission.

### 2.2 Developing Topics and Key Questions

During the first guideline meeting, the key questions for the development of the guidelines were discussed and defined in accordance with the guideline methodology. The key questions cover all clinical, laboratory, diagnostic, and therapeutic aspects that must be addressed during the guideline development process. The guideline group was divided into subgroups, each focusing on specific topics: (I) Clinical Presentation; (IIa) Diagnosis: laboratory tests; (IIb) Diagnosis: imaging; (III) Treatment; (IV) Complications and Long‐Term Management.

### 2.3 Systematic Literature Search

A comprehensive literature review on THD was conducted up to December 2023. This review utilized the PubMed, Cochrane, and Cinahl databases, employing the search terms “Tyrosine Hydroxylase Deficiency” and “THD.” No language or data filters have been applied.

Additionally, reviews on monoamine neurotransmitter deficiencies were searched using PubMed with the search term “_monoamine neurotransmitter disorders_”, limited to reviews in English published from 1990 onwards. The clinical trials registry of the World Health Organization and the National Institutes of Health (NIH) registry were examined. Additionally, reference lists from review articles and significant case series were reviewed for further literature, while members of the guideline group were invited to recommend pertinent book chapters. The flowchart detailing the results of the literature search is available as Additional file: Figure Systematic literature search.

### 2.4 Grading of Evidence and Recommendations

The guideline was developed in accordance with the SIGN framework (SIGN50). In evaluating the quality of evidence and establishing the strength of recommendations, SIGN adheres to the GRADE methodology [6, 7, 8, 9]. The level of evidence of individual studies was rated in a range from 4 (lowest) to 1+ + (highest) (Table 1). Specific outcomes (e.g., effect of a specific drug on hypotonia) were described with a certain level of evidence (very low, low, moderate, or high) for the total body of evidence. Recommendations were rated as strong (for or against), conditional (for or against), or recommendations for further research (Table 2). Additionally, Good Practice Points (GPP) were established based on the clinical expertise of the guideline development team. At least two members of the guideline working group assessed the pertinent literature. Prior to and throughout meetings, the guideline group members received training on standardized literature evaluation employing SIGN/GRADE methodologies. All recommendations were reviewed and discussed to reach a consensus during the group meetings.

**TABLE 1:** Levels of evidence according to SIGN.

Levels of evidence	
1++	High quality meta‐analyses, systematic reviews of RCTs or RCTs with a very low risk of bias
1+	Well conducted meta‐analyses, systematic reviews, or RCTs with a low risk of bias
1−	Meta‐analyses, systematic reviews, or RCTs with a high risk of bias
2++	High quality systematic reviews of case control or cohort studies High quality case control or cohort studies with a very low risk of confounding or bias and a high probability that the relationship is causal
2+	Well conducted case control or cohort studies with a low risk of confounding bias and a moderate probability that the relationship is causal
2−	Case control or cohort studies with a high risk of confounding or bias and a significant risk that the relationship is not causal
3	Non‐analytic studies, e.g., case reports, case series
4	Expert opinion

**TABLE 2:** Forms of recommendations according to GRADE.

Judgment	Recommendation
Undesirable consequences clearly outweigh desirable consequences	Strong recommendation against
Undesirable consequences probably outweigh desirable consequences	Conditional recommendation against
Balance between desirable and undesirable consequences is closely balanced or uncertain	Recommendation for research and possibly conditional recommendation for use restricted to trials
Desirable consequences probably outweigh undesirable consequences	Conditional recommendation for
Desirable consequences clearly outweigh undesirable consequences	Strong recommendation for

## 3 Disclaimer

The aim of this guideline is to improve the care of patients with THD. It is based on the best available evidence from literature. However, due to the extreme rarity of THD as an ultra‐rare condition, the current evidence is comprised of non‐analytical studies and case reports. Thus, many recommendations are based on expert opinion. This guideline is a reliable resource for THD patient caregivers, but it should complement, not replace, informed clinical judgment.

## 4 Part I: Clinical Presentation

### 4.1 Number of Reports; Literature Search

The retrospective analysis of published case reports forms the basis for the description of the clinical symptoms in THD. The analysis was based on 190 individual cases described in the literature and their symptoms before the treatment initiation. Duplicate published cases were identified and excluded where possible; however, it is acknowledged that some duplicates may still be present in the final evaluation. There were considerable differences between the publications analysed regarding the accuracy and depth of detail of the clinical description, for example in the retrospective literature analysis, standardized medical terminology was not employed to describe the clinical symptoms. To improve accuracy, a Human Phenotype Ontology (HPO) term was used for each clinical symptom when possible. Different terms for the same symptom were summarized under one HPO term.

### 4.2 Clinical Phenotype

The phenotypic spectrum of THD in the cases studied ranges from mild to very severe. The cardinal symptoms of THD reflect the lack of dopamine, norepinephrine, and epinephrine in the central nervous system (CNS). The most common clinical signs are not specific for THD, but can also occur in other, sometimes much more common neurological disorders (e.g., cerebral palsy). Nevertheless, some symptoms, such as oculogyric crises (OGC), infantile parkinsonism, and diurnal variations of symptoms with improvement after rest/sleep may indicate an underlying biogenic amine deficiency.

A division of THD into two broad phenotypes has previously been described [3]: Type A refers to an infantile‐onset, progressive, hypokinetic‐rigid syndrome that may or may not include dyskinetic movements and generally shows a positive response to L‐dopa (Dopa‐responsive dystonia or DRD). The type B phenotype is characterized as a complex encephalopathy with neonatal or early infantile onset, presenting a broader range of movement disorders, often accompanied by cognitive impairment. Type B patients tend to be hypersensitive to L‐dopa, experiencing motor fluctuations and dyskinesia at low doses, and may respond poorly to treatment.

The literature review as well as the experiences of the guideline group members suggested a need to re‐evaluate this strict division into two distinct phenotypes.

*R#1 (strong)*: The phenotypic spectrum of THD is a continuum and can range from very mild to severe phenotypes. The previous clinical differentiation in type A and type B should not be applied anymore.

### 4.3 Clinical Patterns

**FIGURE 1:**
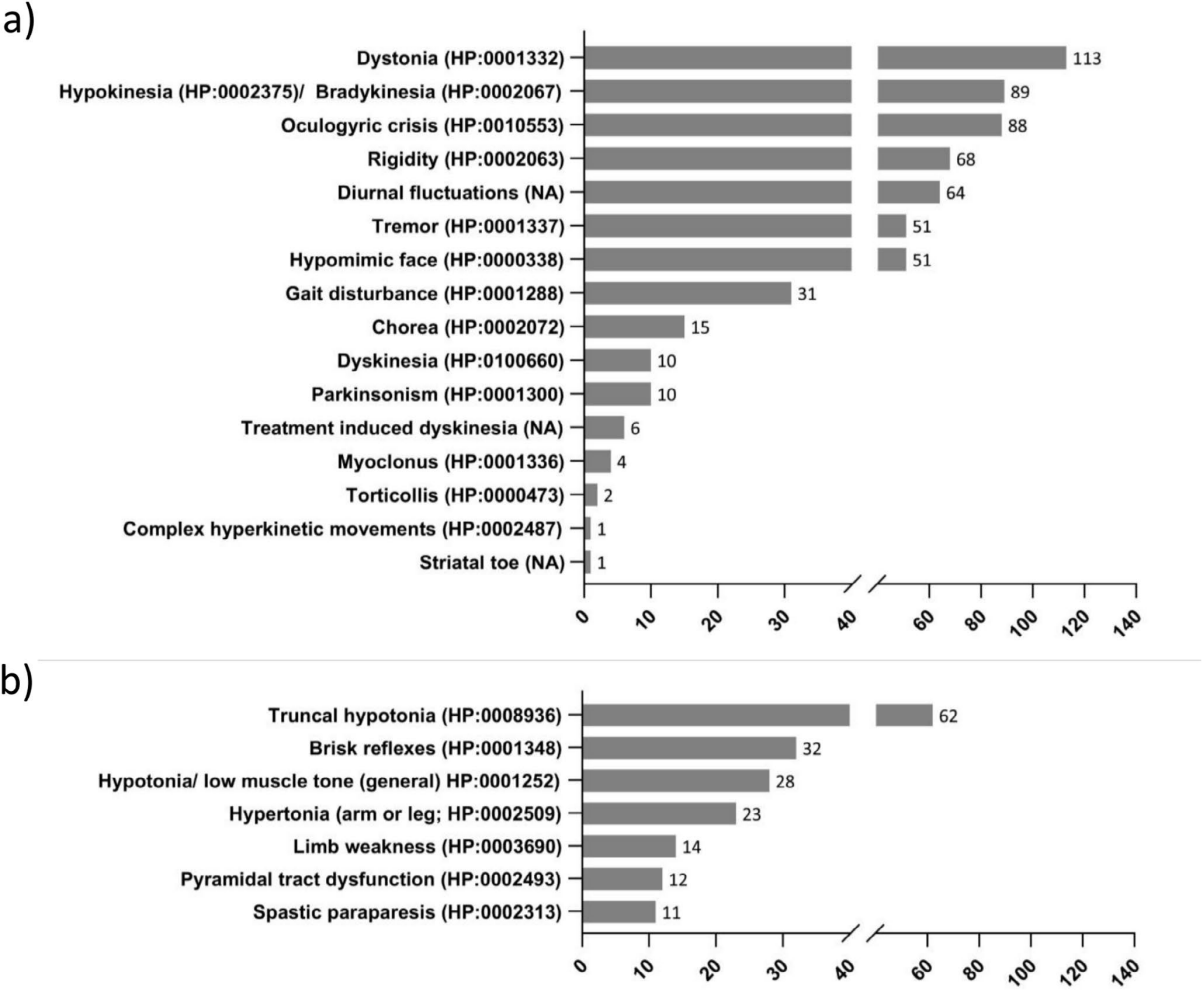
Clinical presentation of THD patients; (a) Movement disorders and extrapyramidal signs, (b) Muscle tone abnormalities (Symptoms are HPO coded where possible, the bar charts show the number of reports of the particular symptom in the available literature).

**FIGURE 2:**
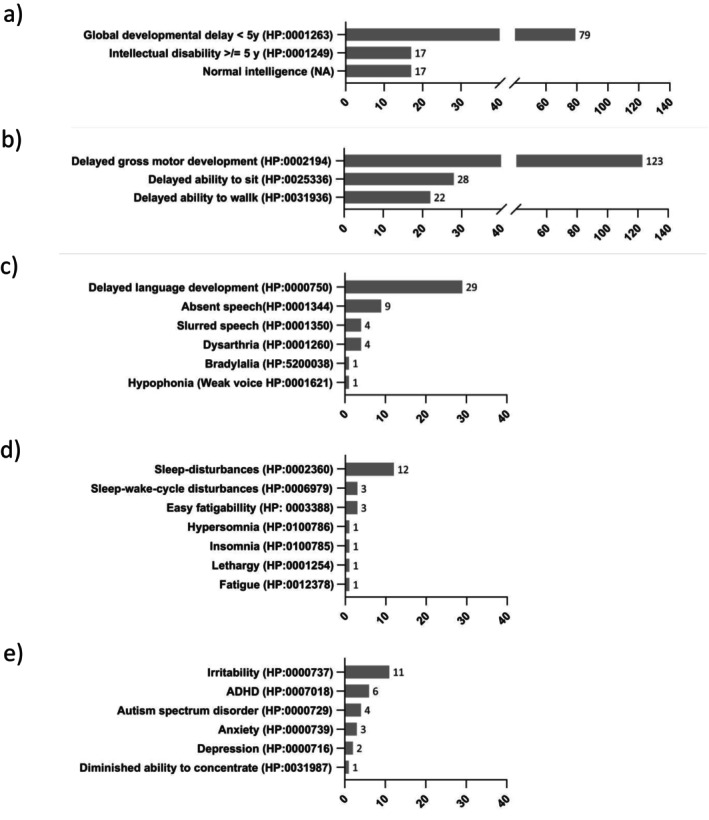
Clinical presentation of THD patients; (a) Cognitive development, (b) Motor milestones, (c) Speech development, (d) sleep disorders and (e) Psychiatric and behavioural symptoms. (Symptoms are HPO coded where possible, the bar charts show the number of reports of the particular symptom in the available literature).

**FIGURE 3:**
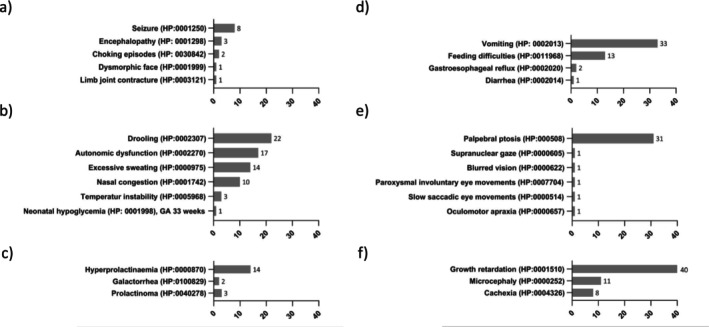
Clinical presentation of THD patients; (a) Further documented neurological symptoms, (b) Autonomic symptoms, (c) Endocrine disorders, (d) gastrointestinal symptoms, (e) Eye related symptoms and (f) Abnormal anthropometric findings. (Symptoms are HPO coded where possible, the bar charts show the number of reports of the particular symptom in the available literature).

**FIGURE 4:**
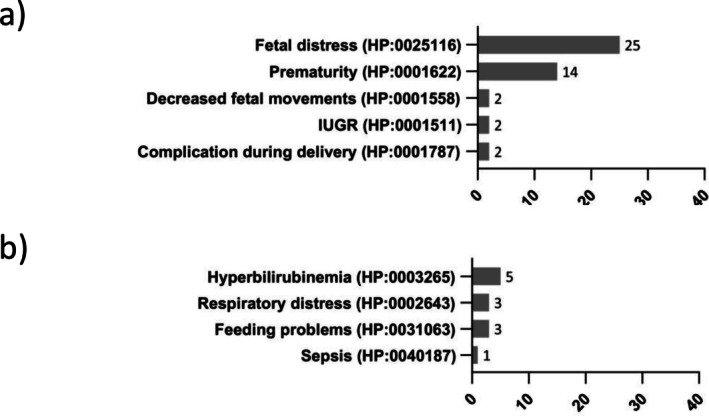
Clinical presentation of THD patients in special situations (a) Pregnancy related symptoms and (b) neonatal disorders (Symptoms are HPO coded where possible, the bar charts show the number of reports of the particular symptom in the available literature).

The various clinical symptoms described are listed in detail in Figures 1, 2, 3, 4. The bar charts show the number of reports of the particular symptom in the available literature. The percentages given in the text below refer to the 190 published TH patients evaluated. As this is an evaluation of published literature, it cannot be ruled out that the symptoms reported for a patient are incomplete.

Movement disorders and delayed gross motor development are the most frequent characteristic of THD. Within the movement disorders, dystonia (HP:0001332, *n* = 113, 59%); hypokinesia/bradykinesia (HP:0002375/HP:0002067, *n* = 89, 47%), rigidity (HP:0002063, *n* = 68, 36%), tremor (HP:0001337, *n* = 51, 27%), hypomimic face (HP:0000338, *n* = 51, 27%), gait disturbance (HP:0001288, *n* = 31, 16%), chorea (HP: 0002072, *n* = 15, 8%), dyskinesia (HP:0100660, *n* = 10, 5%) and parkinsonism (HP:0001300, *n* = 10, 5%) were the most frequently reported symptoms. Movement disorders showed diurnal fluctuation (worsening during the day; subsequent improvement after rest), a characteristic finding in all types of neurotransmitter‐related biogenic amine disorders, in 64 THD cases (34%). OGC (HP:0010553, *n* = 88, 46%) is the most frequently described eye movement abnormality. The other most common symptoms were abnormalities of muscle tone including truncal hypotonia (HP:0008936, *n* = 62, 33%), hypotonia/low muscle tone (general) (HP:0001252, *n* = 28, 15%), hypertonia (arm or leg; HP:0002509, *n* = 23, 12%), limb weakness (HP: 0003690, *n* = 14, 7%), pyramidal tract dysfunction (HP:0002493, *n* = 12, 6%), spastic paraparesis (HP:0002313, *n* = 11, 6%) and brisk reflexes (HP:0001348, *n* = 32, 17%), were listed.

Palpebral ptosis (HP:0000508, *n* = 31) was noted in 16% of THD patients. Further neurological disorders such as seizures (HP:0001250, *n* = 8, 4%) were not common.

Cognitive and language development can be affected by THD with variable severity. A minority of 17 cases (9%) were reported to have normal intelligence, whereas 96 individuals (51%) were documented with cognitive impairments. Divided by age, 79 children (42%) younger than 5 years had global developmental delay (HP:0001263) and 17 patients (9%) aged 5 years and older had intellectual disability (HP:0001249). A delay in language development (HP:0000750) was described in 29 cases (15%), and 9 of the published cases (5%) did not develop any speech (HP:0001344) at all.

*R#2 (strong)*: In cases with unexplained movement disorders (such as dystonia or OGC), alterations in muscle tone (hypotonia/hypertonia), parkinsonism or hypokinetic rigid syndrome and/or developmental delay, THD or other disorders of the biogenic amines should be considered.

### 4.4 Additional Clinical Findings

Mood and behavioural symptoms like ADHD (HP:0007018, *n* = 6, 3%) and irritability (HP:0000737, *n* = 11, 5%) can occur and are likely to be under‐reported in the literature. Autonomic dysregulation, reflecting the disruption of neurotransmitter homeostasis, can be present in THD. Described symptoms consist of drooling (HP:0002307, *n* = 22, 12%), excessive sweating (HP:0000975, *n* = 14, 7%), and nasal congestion (HP:0001742, *n* = 10, 5%). Gastrointestinal disorders can be prominent and include for example vomiting (HP:0002013, *n* = 33, 17%) and feeding difficulties (HP:0011968, *n* = 13, 7%). Endocrine disorders like hyperprolactinemia (HP:0000870, *n* = 14, 7%) can be found in some reported cases. Anthropometric disturbances such as growth retardation (HP:0001510, *n* = 40, 21%), microcephaly (HP:0000252, *n* = 11, 6%), and cachexia (HP:0004326, *n* = 8, 4%) can also occur. Further rare findings are summarized in Figure 3.

It is important to note that the reported symptoms may be more common in THD than suggested, as they are likely underestimated in published cases where clinical phenotypes were less precisely described.

### 4.5 Pregnancy, Birth, and Neonatal Period

Patients with THD may experience problems as early as the prenatal, birth, and neonatal period. During pregnancy, foetal distress (HP:0025116) was reported for 25 published cases (13%), and 14 cases (17%) had a premature birth (HP:0001622) [10]. Since many publications don't focus on the pregnancy, birth, and neonatal period, problems occurring in this very early phase of life might be underreported. At the same time, it cannot be excluded that some of the reported problems occurred independently of THD (Figure 4).

### 4.6 Age of Onset and Age of Diagnosis

The age of onset for signs and symptoms was documented in 154 cases. Among these, 20 cases (13%) presented symptoms already in the neonatal period, while 104 cases (67%) showed disease onset during infancy, and 47 (30%) during childhood. Three cases (2%) remained asymptomatic until adulthood; in one of these cases, symptoms emerged during the patient's pregnancy. The mean age of diagnosis was 7.60 years (median 3.25 years, range 5 months—38 years, 40% missing data).

### 4.7 Prognosis and Prediction of the Disease Course

So far, there is no standard tool to assess disease severity, resulting in evaluation based on the individual judgment of each author. Disease severity evaluation was reported for 83 THD cases, with 21 (25%) classified as mild, 5 (6%) as moderate, and 57 (69%) as severe. There are no reports available on asymptomatic cases. A treatment response was documented in 160 cases. Most patients (88%) experienced a favourable outcome, while the rest demonstrated only partial or poor responses. Additional information can be found in Part III.

### 4.8 Phenotype Correlations With Genotype or Biochemical Phenotype

Based on the literature evaluation, there is no clear genotype/phenotype correlation possible; however, the CSF HVA levels and the response to L‐dopa treatment seem to allow a differentiation of severity. Patients with a severe phenotype had lower HVA levels than those with a mild phenotype and more frequently had dyskinesia following the initiation of L‐dopa and a poor treatment response [3, 11].

*R#3 (for research)*: Further research is required to investigate a genotype/phenotype correlation as well as the correlation of CSF values and clinical phenotype.

## 5 Part IIa: Diagnosis: Laboratory Tests

### 5.1 Key Diagnostic Tests

#### 5.1.1 Lumbar Puncture and CSF Measurements

**FIGURE 5:**
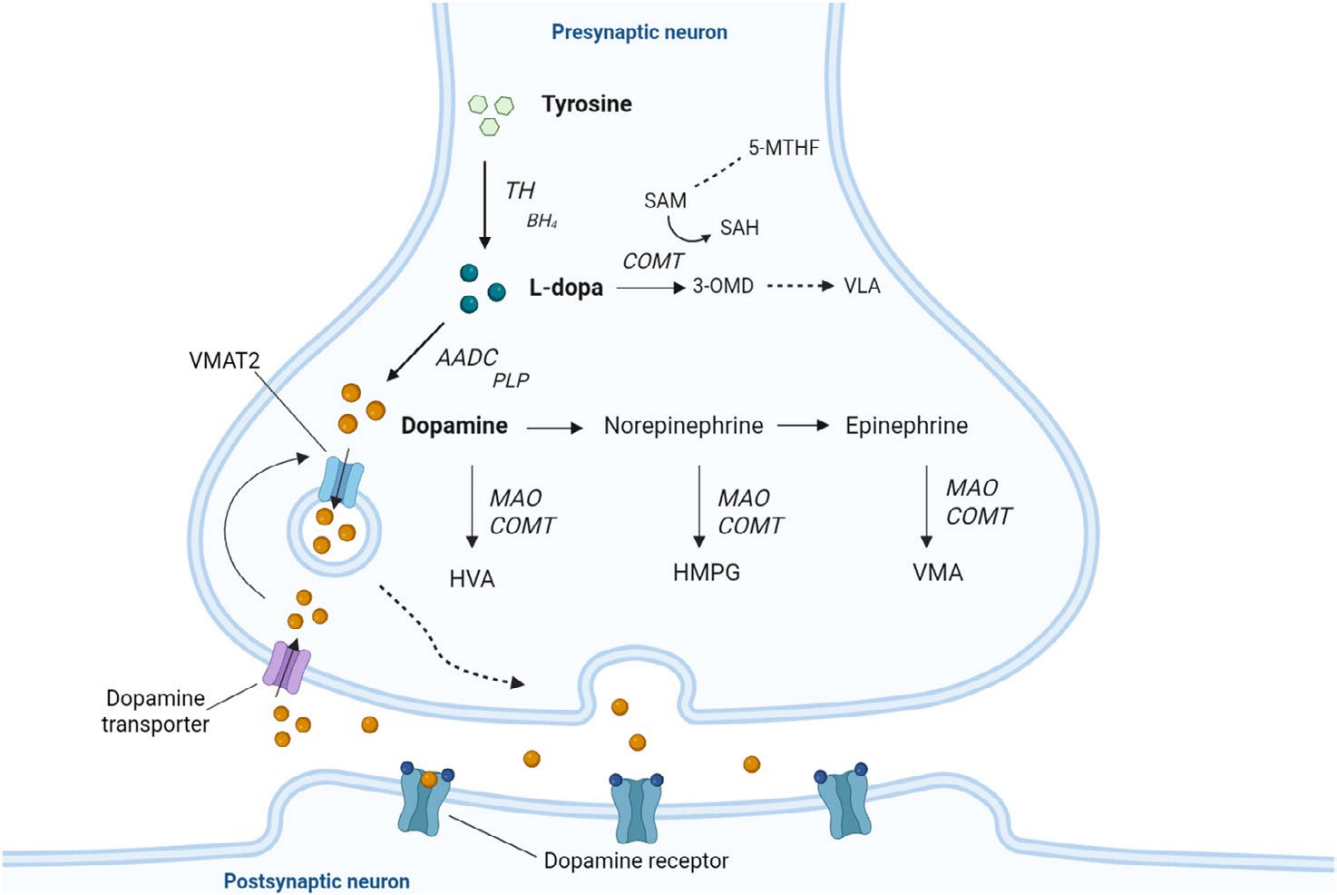
Biosynthesis of dopamine and the metabolic block in tyrosine hydroxylase (TH) deficiency. 3‐OMD, 3‐O‐methyldopa; 5‐HIAA, 5‐hydroxyindoleacetic acid; 5‐MTHF, 5‐methyltetrahydrofolate; AADC, Aromatic l‐amino acid decarboxylase; BH_4_, Tetrahydrobiopterin; COMT, Catechol‐O‐methyl transferase; HVA, Homovanillic acid; L‐dopa, l‐3,4‐dihydroxyphenylalanine; MAO(−I), Monoamine oxidase (inhibitor); PLP, Pyridoxal phosphate; SAH, S‐adenosyl‐homocysteine; SAM, S‐adenosyl‐methionine; TH, Tyrosine hydroxylase; VLA, Vanillactic acid; VMAT2, Vesicular monoamine transporter type 2.

Biogenic amines neurotransmitter disorders can be identified by detecting abnormal levels of neurotransmitters and their metabolites in the CSF. Each specific pattern of these biochemical markers suggests a different disorder. Tyrosine hydroxylase catalyses the initial and rate‐limiting step in the synthetic pathway of dopamine, that is, the conversion of the amino acid L‐tyrosine to levodopa (L‐3,4‐dihydroxyphenylalanine) in the presence of the cofactor tetrahydrobiopterin (BH_4_). Levodopa is further processed to dopamine by L‐aromatic amino acid decarboxylase (AADC) and its cofactor pyridoxal‐5′‐phosphate (PLP) and subsequently metabolized to norepinephrine by dopamine‐β‐hydroxylase. Their degradation products HVA and MHPG, respectively, are thus expected to be decreased in THD. Serotonin is produced in two consecutive steps from tryptophan, which is converted by tryptophan hydroxylase to 5‐hydroxytryptophan (5‐HTP) later forming serotonin by the action of AADC. The levels of serotonin and related metabolite [5‐hydroxyindoleacetic acid (5‐HIAA)] are preserved in THD (Figure 5).

The diagnostic hallmarks of the CSF biochemical profile in THD include (1) low HVA and normal 5‐HIAA, (2) decreased HVA/5‐HIAA ratio, (3) low MHPG, and (4) normal pterins, reflecting the metabolic block at the level of tyrosine hydroxylase (Figure 5). To prevent preanalytical influences on CSF collection and handling, lumbar puncture should be performed strictly according to standardized procedures and local laboratory protocols to ensure correct interpretation of the results ([12]).

Homovanillic acid results were obtained for 52 individuals, all of whom demonstrated low levels (100%). 5‐HIAA levels were normal in 43 out of 48 patients (90%) and low in 5 patients [13, 14, 15, 16]. Among these five patients, the HVA/5‐HIAA ratios were as follows: 0.7 [15]; 0.18 [14]; 0 and 0.18 [16]; and 0.38 [13]. All 43 patients showed a decreased HVA/5‐HIAA ratio. Low MPHG values were found in 19 out of 20 patients (95%). One patient was found to have normal MPHG levels while the other relevant parameters clearly pointed to THD (low HVA, normal 5‐HIAA, and low HVA/5‐HIAA ratio [17]). It should be noted that MPHG is not routinely measured in clinical settings in most CSF laboratories but rather reserved for research purposes. 3‐OMD was reported as not elevated in any patient (low or normal in 21 out of 21 patients tested). 5‐HTP levels were typically normal, although measurement was rarely performed in patients with THD. Not elevated levels of 3‐OMD and 5‐HTP may aid in distinguishing THD from AADCD (aromatic L‐amino acid decarboxylase deficiency) [18].

Pterin analysis in THD revealed normal biopterin and neopterin levels in 15 out of 19 patients (79%). Two patients had borderline low biopterin and neopterin levels [14, 19], two others had borderline low neopterin [13, 14]. These findings may assist in differentiating THD from tetrahydrobiopterin deficiencies, which exhibit specific patterns of pterin alterations [20].

The results gathered from individual case reports or case series are in concordance with a large iNTD registry‐based analysis of 44 patients with THD also reporting decreased HVA and HVA/5‐HIAA ratio in almost all samples, typically normal 5‐HIAA and 5‐HTP levels in the majority and normal pterin levels in a similar proportion of patients [10].

It must be noted that numerous other diseases affecting the CNS exhibit secondary alterations in neurotransmitter levels, for example, epilepsy, epileptic encephalopathies, mitochondrial disorders, or other inborn errors of metabolism, hypoxic–ischemic encephalopathies, leukodystrophies, neurogenetic syndromes, acquired CNS injuries, infections, and many others [12, 21]. It is therefore very important not to base the interpretation of biogenic amines and the diagnosis of THD solely on HVA levels, but to also include the HVA/5‐HIAA ratio in the evaluation.

#### 5.1.2 CSF Folate

Long‐term L‐dopa therapy may lead to excessive O‐methylation of L‐dopa to 3‐OMD, which may deplete the methyl donor groups including S‐adenosylmethionine (SAM) and 5‐methyltetrahydrofolate and lead to secondary folate deficiency [22]. Folate deficiency was evaluated in 6 patients, with 2 patients having low levels [14]. A patient was described with initially normal CSF levels that decreased after L‐dopa/carbidopa therapy. The patient improved significantly after supplementing the low folate levels [23]. Analysis of 5‐MTHF should thus be included in the CSF analysis, if performed. However, data on 5‐MTHF status in patients with THD on long‐term L‐dopa/DCI therapy are missing, and further research is needed.

*R#4 (strong)*: The characteristic CSF biochemical pattern of THD consists of low HVA and normal 5‐HIAA with a low HVA/5‐HIAA ratio and normal pterins.

*R#5 (conditional)*: 5‐MTHF in CSF can be decreased in THD. Testing for CSF 5‐MTHF levels may be considered.

*R#6 (research)*: Further research is required to determine the folate status of patients with THD both initially and during long‐term L‐dopa therapy.

*R#7 (GPP)*: CSF measurement should always include routine diagnostic analyses to exclude other causes in the differential diagnosis.

*R#8 (GPP)*: CSF sampling and handling should strictly follow standardized procedures to ensure valid and reliable results.

### 5.2 Genetic Testing

THD is an autosomal recessive disorder. A total of 105 pathogenic variants have been reported in the TH gene, including 81 missense/nonsense, 8 splicing, 3 regulatory, 4 small deletions, 1 small insertion, 1 indel, 6 gross deletions, and 1 complex rearrangement. An additional 19 likely pathogenic variants have been described, including 15 missense/nonsense and 4 splicing variants (Human Gene Mutation Database—HGMD Professional 2024.2). Common variants have been reported in some populations, for example, the Dutch and Greek [24].

DNA is sampled from the peripheral blood. TH gene analysis using Sanger sequencing was reported in approximately two‐thirds of patients, while multi‐gene panel and whole‐exome sequencing were performed in the remaining one‐third.

The increasing availability of multi‐gene panel sequencing or next‐generation sequencing techniques offers an effective approach, particularly in patients with equivocal biochemical findings or atypical clinical presentation. Given the reduced “time to result” of these methods, they may in fact be the first step in the diagnostic evaluation of a patient with clinical findings suggestive of THD.

*R#9 (strong)*: Genetic testing to identify biallelic pathogenic variants in the TH gene should be performed when THD is suspected.

### 5.3 Concluding Statements Regarding the Key Diagnostic Tests

The essential diagnostic tests for THD are:

- CSF: Low HVA and normal 5‐HIAA with low HVA/5‐HIAA ratio and normal pterins.
- Genetic analysis verifying pathogenic variants in the TH gene.

#### 5.3.1 Other Diagnostic Tests

We reviewed the utility of additional diagnostic tests described in the literature: TH activity, urine neurotransmitter metabolites, urine pterins, and blood prolactin.

#### 5.3.2 TH Activity Measurement

The protein expression of TH is tissue‐specific (mainly adrenal medulla, peripheral nerve fibers, and certain brain regions, e.g., nucleus caudatus) [25]. The TH enzymatic activity is, therefore, currently not applicable for diagnostic purposes. There are no studies available that analyze TH enzymatic activity in patients with THD.

*R#10(strong)*: TH enzymatic activity measurement is currently not recommended for diagnostic purposes.

##### 5.3.2.1 Urine Measurement of Neurotransmitter Metabolites—Dopamine, Epinephrine, Norepinephrine

Urine levels of dopamine, epinephrine, and norepinephrine were analysed in only a small proportion of patients with THD [26]. Urinary dopamine levels showed decreased values in 2/10 patients (20%) [22, 27]. Urinary epinephrine excretion was low in 1/10 patients (10%) [19]. Urinary norepinephrine was decreased in 7/10 patients (70%) and normal in only 3 patients [22]. Overall, urine measurement of neurotransmitter metabolites dopamine, epinephrine, and norepinephrine is not a reliable marker to diagnose THD. Furthermore, these parameters may be influenced by dietary intake.

*R#11 (strong)*: Urinary dopamine, epinephrine, and norepinephrine are not reliable diagnostic tools and should not be used to diagnose THD.

##### 5.3.2.2 Urinary Measurement of Pterins

From the pathophysiologic point of view, pterins are not expected to be altered in THD. Urine analysis of pterins (biopterin and neopterin) was performed in 13 patients and yielded normal results in 12 out of 13 patients (92%) with one slightly lower level of biopterin in one [28]. The measurement of pterins in urine can, however, be considered as part of a general diagnostic work‐up in patients with suspected neurotransmitter disorders.

*R#12 (strong)*: Urinary pterins are normal in THD and should not be used to diagnose THD.

##### 5.3.2.3 Urinary Measurement of Vanillactic Acid

Vanillactic acid (VLA) in urine, the L‐dopa degradation product, was measured in 9 patients and yielded normal results in all [17, 22, 29]. Therefore, it cannot serve as a diagnostic marker for THD. The measurement of VLA in urine can, however, be considered as part of a general diagnostic work‐up in patients with suspected neurotransmitter disorders, and if elevated, may point towards AADC deficiency [18].

*R#13 (strong)*: Urinary vanillactic acid is normal and should not be used to diagnose THD.

#### 5.3.3 Blood Tests: Prolactin

Dopamine serves as an inhibitor of prolactin secretion under normal conditions. Low levels of dopamine in THD are thereby expected to be accompanied by increased prolactin release. Prolactin blood levels were examined in 24 patients and were elevated in 19 of them (79%) and normal in 5 patients [16, 30]. However, since prolactin secretion follows physiologically a diurnal pattern and may be affected by numerous causes, its normal levels do not rule out the diagnosis of THD.

Hyperprolactinaemia‐related symptoms, such as menstruation irregularities, pubertal delay/arrest, galactorrhoea, or heterogeneity/hyperplasia, or adenoma of the pituitary gland, have recently been described in patients with neurotransmitter disorders, including THD, with a median age at diagnosis of 16 years [31]. It appears rational to screen for prolactin levels and hyperprolactinemia‐related comorbidities, especially during/after puberty.

*R#14 (research)*: Prolactin may be elevated in THD because of dopamine deficiency, but normal levels do not exclude THD. It cannot be utilized to diagnose or exclude THD. Additional research is suggested on blood prolactin levels as both a diagnostic marker for THD and as a tool for monitoring treatment response.

*R#15 (conditional)*: Blood prolactin should be monitored during and after puberty to screen for hyperprolactinemia as comorbidity.

## 6 Part IIb: Diagnosis: Imaging, Electroencephalography

### 6.1 Imaging

#### 6.1.1 Brain MRI (Magnetic Resonance Imaging)

Brain MRI abnormalities are less common and more variable in patients with THD compared to patients with other monoamine neurotransmitter disorders [32]. Brain MRI was performed in 79 patients with THD and was reported normal in 66 of them (84%). The most common pathology in the remaining 13 patients was brain atrophy in 8/79 patients (10%), presumably as a common non‐specific finding in patients with chronic neurological disorders. Five other patients displayed non‐specific MRI abnormalities.

A large iNTD registry analysis of brain MRI of patients with monoamine neurotransmitter disorders confirms that fewer than half of the patients have changes on brain MRI, and brain atrophy is identified as the most common pathology [32].

Although not specifically diagnostic for THD, brain MRI is typically performed as part of the initial diagnostic algorithm in any patient who presents with neurodevelopmental abnormalities. It should also be considered in patients with THD who develop unexpected clinical symptoms.

*R#16 (strong)*: Routine imaging of the brain is not required to diagnose THD.

*R#17(GPP)*: Indication for MRI should be based on clinical/neurologic evaluation. If the symptoms do not align with the THD phenotype, an MRI may be considered.

### 6.2 Other Imaging Modalities

#### 6.2.1 Nuclear Imaging

Brain nuclear imaging was performed in 10 patients using various modalities focusing on brain neurotransmitters (dopamine) or dopamine transporter levels (Single‐photon emission tomography (SPECT), MR spectroscopy, Positron Emission tomography (PET)). The results were normal in nine patients. Only 1 patient was reported to have slightly decreased dopamine type 2 receptor binding in the striatum [33].

*R#18 (Conditional)*: Nuclear imaging techniques are not routinely indicated to make a diagnosis of THD.

### 6.3 Electroencephalography (EEG)

EEG was altogether performed in 39 patients and was described as normal in 37 of them (95%). One patient was reported to have intermittent bursts of sharp waves on his EEG [34]; the other displayed nonspecific generalized dysrhythmia [29]. THD is not accompanied by any specific EEG abnormalities. Nevertheless, in case of a clinical suspicion of epilepsy, EEG should be performed as a part of good clinical practice. EEG also helps to differentiate epileptiform activity from OCG, frequently accompanying THD.

*R#19 (strong)*: Routine EEG is not required to diagnose THD.

*R#20 (GPP)*: Clinical and technical examinations (EEG, ECG) and consultations should be conducted in case of suspected epilepsy and/or psychiatric or psychological abnormalities, respectively.

## 7 Part IIc: Diagnosis: Prenatal and Newborn Screening

### 7.1 Prenatal Screening

Early diagnosis allowing for timely treatment is of utmost importance for patients with THD. Targeted testing may be performed in pregnancies following an index patient with a confirmed diagnosis of THD.

Prenatal screening was reported in two families with an already affected older sibling [35, 36]. Mutation analysis of chorionic‐villi samples was successfully conducted in both cases.

*R#21 (strong)*: Prenatal screening should be offered to high‐risk families with a previously diagnosed child with THD, the method of choice being genetic testing.

### 7.2 Newborn Screening

There is no literature evidence on newborn screening (NBS) in THD.

THD is currently not subject to traditional biochemical newborn screening. The potential application of molecular genetic newborn screening is being investigated for its suitability in the context of THD.

*R#22 (research)*: THD is not a target disease for traditional biochemical NBS. A research recommendation is given to investigate the feasibility of genetic NBS for diagnosing THD.

## 8 Part III: Treatment

### 8.1 Available Evidence

The THD guideline group reviewed data about treatment in a total of 65 studies: 35 case reports and 30 case series (160 patients in total). We included studies that contained sufficient details about dosage, benefits, side effects, and treatment outcomes to inform judgments about medication use. We excluded 30 of the 190 total cases described in the literature from this analysis due to insufficient information.

The overall quality of the evidence was moderate to low. Limitations included the type of publications available, incomplete detail provided about clinical outcomes in many cases, and lack of standardized descriptions of outcomes between studies. In the case of L‐dopa, though, these limitations were counterbalanced by the large treatment effect described in most patients across many studies, and the detailed description of treatment and response provided in a few. Evidence for or against the use of medications other than L‐dopa is much scarcer in the literature, and thus recommendations are influenced by expert opinion here.

## 9 Part IIIa: Treatment: First‐Line Treatment

### 9.1 L‐dopa

L‐dopa is the precursor of dopamine and is administered to correct the dopamine deficiency state in THD [37]. It is typically administered in combination with a peripheral DCI, either carbidopa or benserazide, to reduce systemic side effects such as nausea and increase L‐dopa delivery to the brain.

Most reported patients had a good response to treatment (141/160, 88%), while nine patients had a partial response. Positive effects included resolution of or improvement in dystonia, hypokinesia, OGC, and autonomic dysfunction (ptosis, sweating), accompanied by motor developmental progress in patients with early infantile onset disease associated with developmental delay. The speed and degree of response were quite variable across patients. At the mild end of the disease spectrum, characterized by patients with late‐infantile or childhood onset of dopa‐responsive dystonia, symptoms tended to respond rapidly to low doses of L‐dopa, and normalization of motor function was reported in many cases. At the severe end of the disease spectrum, characterized by symptom onset in early infancy and little or no attainment of motor milestones in the first 6 months of life, very gradual dose titration was often required, and clinical improvements occurred over a period of months to years. Objective measures of developmental and functional outcomes in patients with early infantile onset disease were described in a small number of studies. Both motor and intellectual functional outcomes varied widely, from normal to severe disability [5, 16, 38, 39, 40, 41].

A poor response to L‐dopa was described in just 10 patients [3, 16, 17, 27, 39, 42, 43, 44] who had a severe phenotype with symptom onset before age 6 months, and a tendency toward later treatment initiation (median age 4.0 years, range 1.9–19 years) compared to those with symptom onset before age 6 months who had a good treatment response (*n* = 58, treatment initiation at median age 1.7 years, range 0.4–13 years). This suggests that early initiation of treatment is beneficial but may not be the only factor that determines the long‐term outcome given the significant overlap in the range of treatment initiation ages between these two groups. The significant variability in primary disease severity also likely plays a role.

*R#23 (strong)*: L‐dopa in combination with a DCI (carbidopa or benserazide) should be used as the primary treatment for THD.

The starting dose, rate of dose titration, and maintenance dose of L‐dopa varied considerably between patients. One key factor that may influence dosing is the tolerability of symptoms that may emerge soon after treatment initiation. The most common of these is involuntary movements (dyskinesia), which were reported in 36 patients. Irritability or behaviour disturbance (*n* = 8) and insomnia (*n* = 4) have also been described. It has been proposed that super sensitization of dopamine receptors contributes to the development of L‐dopa‐induced dyskinesia in patients with THD, owing to chronic dopamine deficiency during a critical period of neuromotor development [11]. Additionally, the role of the non‐regulated release of exogenously derived dopamine, which leads to large and intermittent fluctuations in the extracellular levels of dopamine in the brain, is a potential key factor in the development of dyskinesia. The latter is supported by the improvements in dyskinesia by increasing the frequency of L‐dopa dosing that facilitates more steady levels of dopamine and also by the favourable effect of selegiline that, by inhibiting dopamine degradation, results in a more continuous exposure to dopamine in the striatum (see next section) [3, 5]. Nausea and vomiting are another potential side effect of L‐dopa, although they were reported rarely (*n* = 4).

Most patients who experienced dyskinesia were able to continue L‐dopa therapy with some combination of a temporary decrease in the dose, increased dosing frequency, and very gradual dose titration. The addition of selegiline was also reported to be beneficial in 15 cases (see Supplementary Dopaminergic Treatment, below). Three patients discontinued L‐dopa due to intolerable dyskinesias and/or irritability [5, 19, 39].

*R#24 (strong)*: L‐dopa/DCI should be started at a low dose and titrated gradually, as tolerated.

*R#25 (strong)*: Dyskinesias are expected to occur at treatment initiation as a reflection of brain dopamine hypersensitivity. This may be managed by (1) temporarily lowering the total daily dose, and/or (2) dividing the medication into more frequent, smaller doses, and/or (3) slowing the rate of dose increases.

*R#26 (strong)*: Dopaminergic medication should be taken life‐long and adjusted according to weight gain during childhood and the occurrence of side effects.

A detailed summary of the L‐dopa/DCI treatment and suggested dose recommendations and their increases can be found in Table 3.

**TABLE 3:** First‐line treatment for THD: L‐dopa dose recommendations according to phenotype.

Phenotype	Starting dose	Rate of increase	Typical maintenance dose	Maximum reported dose	Clinical notes
Infantile dystonia‐parkinsonism	0.5–1 mg/kg/d divided into 3–4 doses	0.5 mg/kg/d every 2–4 weeks	2–10 mg/kg/d	35 mg/kg/d or 800 mg/d	Dyskinesia expected at treatment initiation. If dyskinesia is tolerable, persist with gradual dose titration. If bothersome, reduce total daily dose, divide into smaller/more frequent doses, and slow rate of titration Treatment goal: resolution of symptoms (dystonia, OGC) and observable developmental progress
Dopa‐responsive dystonia	1–2 mg/kg/d divided into 2–3 doses	1 mg/kg/d every 1–2 weeks	2–7 mg/kg/d	500 mg/d	Treatment goal: no dystonia recurrence with full range of physical activity. Doses above 500 mg/day may be used if needed.

The most used ratio of L‐dopa to DCI is 4:1, although the 10:1 formulation is also available. If a patient experiences nausea and vomiting with L‐dopa treatment, the ratio of L‐dopa to DCI becomes an important consideration. The risk of these side effects is lower with the 4:1 formulation compared to the 10:1 formulation. The lower dose of DCI in the 10:1 formulation leads to increased systemic conversion of L‐dopa to dopamine, which may induce the side effects of nausea and vomiting.

## 10 Part IIIb: Treatment: Supplementary Dopaminergic Treatment

### 10.1 Monoamine Oxidase Inhibitors (MAO Inhibitors)

MAO inhibitors prevent the breakdown of monoamines, thereby increasing their availability in the synaptic cleft. There are two main types of MAO enzymes in humans: MAO‐A is involved in the metabolism of dopamine, norepinephrine, and serotonin, while MAO‐B primarily metabolizes dopamine.

The effect of MAO inhibitors in THD was described for 20 cases in 14 studies ([3, 16, 17, 19, 27, 33, 39, 40, 45, 46, 47, 48, 49, 50]). The MAO inhibitor used in almost all cases was selegiline, an irreversible selective MAO‐B inhibitor (*n* = 19), and in one it was not specified ([27]). No studies were found on other MAO inhibitors like tranylcypromine, phenelzine, or rasagiline.

Most of the studies described the effect of MAO inhibitors in combination with L‐dopa. In two patients, it was given as monotherapy sometime in the course of their disease [19, 39]. In one patient, selegiline and L‐dopa were given in combination with either a dopamine agonist or a catechol O‐methyl transferase (COMT) inhibitor [40].

The majority of patients tolerated the MAO inhibitor, except for three patients ([27, 39, 49]) who manifested adverse effects (hyperkinesia, hypertonia, sleep disturbance).

Except for three patients ([19, 27, 39]), the majority of studies described an improvement in at least one clinical endpoint (e.g., motor function, dystonia, OGC). In 3 studies, the authors described patients with hypersensitivity to L‐dopa manifesting with dyskinesia, in which the introduction of selegiline, in addition to improving motor function, allowed further increases of L‐dopa dose [16, 17, 33, 40, 45].

*R#27 (conditional)*: MAO inhibitors can be considered subsequently as a supplementary treatment in THD in combination with L‐dopa when upward titration of the L‐dopa dose is limited by dyskinesia and/or adverse effects (irritability, insomnia, nausea). Selective MAO‐B inhibitors are preferred. The availability of the drug should guide its use.

Dose recommendations are described in Table 4.

**TABLE 4:** Supplementary treatment for patients with THD and Infantile Dystonia‐Parkinsonism.

Mechanism of action	Drug^a^	Starting dose	Rate of increase	Typical maintenance dose	Max dose	Clinical notes
MAO inhibitors	Selegiline	0.1 mg/kg/d, divided into 2 doses, morning + afternoon	0.1 mg/kg/d every 2 weeks	0.1–0.3 mg/kg/d	0.3 mg/kg/d or 10 mg/d	Indications: difficulty tolerating L‐dopa dose escalation, symptom fluctuation, inadequate response to L‐dopa + DCI alone. May cause sleep disturbances, best taken in the morning, afternoon, or at lunchtime. Attention: Orally disintegrating forms need lower doses due to bypassing liver metabolism
COMT inhibitors	Entacapone			4 mg/kg/d		Indications: inadequate response to L‐dopa + DCI alone In many countries, licensed for adults only. ATTENTION: When used with L‐Dopa/DCI; consider reducing L‐Dopa dose by 10%–30%.
	Tolcapone			1.5 mg/kg/d		
Multiple: NMDA glutamate receptor antagonist, increase of dopamine release, Block of dopamine reuptake	Amantadine	4–6 mg/kg/d, divided into 2–3 doses		3 mg/kg/d	20 mg/kg/d	Indications: difficulty tolerating L‐dopa dose escalation Attention: Contraindicated within 14 days of intranasal influenza vaccine. Intramuscular flu vaccine permitted during treatment. Risk of heat stroke—use caution in hot weather. Dose adjustment may be necessary in cases of kidney impairment.

^a^ The drugs listed in this table are those reported in the THD literature. # Leuzzi 2017.

Selegiline can cause insomnia as a side effect, and the risk of this is diminished by administering the doses in the morning and afternoon while avoiding evening doses.

Despite never being reported in the THD literature, caution is advised when combining MAO inhibitors with serotonin reuptake inhibitors, as this combination could increase serotonergic effects, potentially leading to serotonin syndrome.

### 10.2 Dopamine Agonists (DA)

Dopamine agonists exert their effects through the direct stimulation of postsynaptic dopamine receptors. They can be ergot‐derived (bromocriptine, cabergoline, pergolide) or non‐ergot‐derived, including pramipexole, ropinirole, rotigotine (transdermal patches), and apomorphine (subcutaneous). Ergot‐derived DAs are linked to fibrous complications (pulmonary, retroperitoneal, cardiac), though bromocriptine has a lower risk. Non‐ergot‐derived DAs have a very low risk of fibrotic complications and are preferred in clinical practice. The use of DAs has been described in a few patients [3, 33, 40, 49]. Bromocriptine and pramipexole are the most frequently used. Ropinirole and rotigotine patches have been used in single patients [40, 49].

Adverse effects were frequent, including irritability, sleep disturbance, and choreic status with pramipexole [33], akinetic status with rotigotine [40], and peripheral edema with ropinirole [49]. Most studies described either limited or no beneficial effect.

*R#28 (conditional)*: Dopamine agonists may not be used as standard treatment in THD as it is unclear that the benefits outweigh the side effects. They may be considered if other treatment options remain unsuccessful.

If treatment with DA is considered, non‐ergot derived DAs are preferred. If bromocriptine is used, cardiac screening before and during treatment is indicated because of the potential risk of cardiac valvular fibrosis. Cabergoline and pergolide should not be used because of the high risk of fibrotic complications.

### 10.3 Amantadine

Amantadine is a weak antagonist of the NMDA‐type glutamate receptor, increases dopamine release, and blocks dopamine reuptake. It is used for the management of L‐dopa induced dyskinesia in patients with Parkinson's disease.

In the literature, three patients with THD and L‐dopa induced dyskinesia were treated with Amantadine [5, 23]. All patients responded well to treatment and did not have adverse effects. In two patients, the introduction of amantadine allowed further increases of L‐dopa dose.

*R#29 (conditional)*: Amantadine can be considered as a subsequent supplementary treatment in combination with L‐dopa to manage dyskinesia and facilitate up‐titration of the L‐dopa dose.

### 10.4 Catechol‐O‐Methyl Transferase (COMT) Inhibitors

COMT inhibitors prevent the breakdown of monoamines, thereby increasing their availability in the synaptic cleft. Treatment with COMT inhibitors concurrently with L‐dopa and selegiline was reported in a single patient [40]. No adverse effects were reported, and the response to treatment could not be determined since the patient was on multiple drugs. Although current evidence is restricted to a single documented case, the application of these agents in related disorders offers a reasonable basis for their consideration in this setting [20].

*R#30 (conditional)*: COMT inhibitors can be considered as supplementary treatment in THD.

## 11 Part IIIc: Treatment: Symptomatic Treatment

### 11.1 Symptomatic Treatment of Motor Manifestations

Symptomatic treatment for the management of motor symptoms, mainly dystonia, may be used; however, these treatments are generally not needed once a patient is established on primary L‐dopa therapy.

### 11.2 Anticholinergic Drugs

Anticholinergic drugs such as trihexyphenidyl, benztropine, and biperiden are frequently used to manage dystonia and parkinsonism. Their mechanisms of action are believed to involve the modulation of imbalances between dopaminergic and cholinergic pathways.

Several patients with THD received anticholinergics in combination with other agents ([27, 39, 48, 49, 51, 52]). Seven patients received trihexyphenidyl, 6 biperiden, and it was not specified in 1. The effect of anticholinergics was described in two patients; one showed improvement of rigidity and OGC [39], and the other had little benefit [52]. In most reports, the anticholinergic had been given before the diagnosis of THD was confirmed.

Typical anticholinergic side effects (dry mouth, dry eye, blurred vision (pupil dilation), constipation, urinary retention, reduced sweating) were not reported in THD.

*R#31 (conditional)*: Anticholinergic agents may be considered as a symptomatic additional treatment for dystonia in THD. In patients who respond well to L‐dopa, anticholinergic agents can be discontinued once symptoms are well controlled with L‐dopa treatment.

### 11.3 Benzodiazepines

Benzodiazepines enhance the inhibitory activity of gamma‐aminobutyric acid (GABA)_A_ receptors in the CNS and can be used for the management of dystonia.

The use of benzodiazepines was reported in only three patients ([19, 27, 48]). In two patients, clonazepam ameliorated their paroxysmal events and OCGs [19, 48].

*R#32 (GPP)*: Benzodiazepines may be considered as a symptomatic additional treatment for dystonia in THD.

### 11.4 Baclofen

Baclofen is an agonist of (GABA)_B_ receptors in the central and peripheral nervous system and is used for the management of spasticity.

In the literature, there is only one case series in which three patients with THD received baclofen in combination with other agents. No clear description of its clinical effect is provided [48].

*R#33 (GPP)*: Baclofen may be considered a symptomatic additional treatment of spasticity in THD.

### 11.5 Anti‐Seizure Medications

Several patients with THD were treated with anti‐seizure medications because their OGC were interpreted as seizures. One patient showed improvement of OGC and dystonic crisis with lamotrigine [19]. Another patient did not respond to valproic acid [30]. One patient received phenytoin for the treatment of febrile seizures ([27]). In the series of Pons et al. [5], one patient was treated with vigabatrin and levetiracetam, another with phenobarbital, and another had received phenobarbital, valproic acid, vigabatrin, carbamazepine, phenytoin, and topiramate. None of these patients responded to the antiepileptic treatment.

*R#34 (GPP)*: Epileptic seizures are not a cardinal clinical symptom of THD and should be distinguished from OGC or dystonic jerks by using reliable diagnostic approaches. If required, any anti‐seizure medication can be used according to the specific indications for different seizure types.

### 11.6 Symptomatic Treatment of Behavioural and Psychiatric Manifestations

Psychiatric and behavioural manifestations have been reported in some patients with THD; however, motor symptoms are generally the focus of treatment. Determining whether a behavioural symptom represents an adverse effect of L‐dopa administration, or a manifestation of primary catecholamine deficiency can sometimes be challenging, highlighting an unmet need in understanding this condition and developing effective treatments [53].

A broad spectrum of medications is available for the symptomatic management of these manifestations, including antidepressants, stimulants, anxiolytics, and mood stabilizers, all of which may be beneficial for patients with THD. Healthcare providers should adhere to age‐ and dose‐specific guidelines when managing these symptoms.

Although there are no absolute contraindications for the use of selective serotonin reuptake inhibitors (SSRI), serotonin‐norepinephrine reuptake inhibitors (SNRI), or stimulants in THD, caution is recommended due to their effects on monoamine neurotransmitter homeostasis and the potential for exacerbated adverse effects. Dopamine receptor blocking antipsychotic medications should be avoided in THD due to their antidopaminergic effects.

### 11.7 Symptomatic Treatment of Sleep Disturbances

Sleep disturbances have also been reported in some patients with THD, although their management has not been well documented in the THD literature. In some cases, these disturbances are related to the adverse effects of dopaminergic treatment, and in such instances, the dosing of dopaminergic medications can be adjusted accordingly. For cases not related to dopaminergic treatment, management with melatonin may be considered.

## 12 Part IIId: Treatment: Other Metabolic Treatment

### 12.1 Folinic Acid

As raised above, secondary cerebral folate deficiency may develop in patients with THD treated chronically with L‐dopa.

Levels of CSF 5‐MTHF were normal in one patient with THD prior to L‐dopa treatment, and they were low after several months on treatment [23]. The patient was started on folinic acid at 1 mg/kg/day and showed improvements in motor and cognitive function [23]. This effect cannot be attributed specifically to the folinic acid supplementation since the patient was treated with L‐dopa at the same time.

*R#35 (Conditional)*: Supplementation with folinic acid can be considered and is clearly recommended when 5‐MTHF in CSF is low.

*R#36 (Research)*: Further research on 5‐MTHF is needed to be able to give strong recommendations on folinic acid supplementation and 5‐MTHF follow‐up in THD.

### 12.2 Protein‐Redistribution Diet

In order to optimize the L‐dopa efficacy, a protein‐redistribution diet (normal protein content exclusively for dinner) together with intake of special low‐protein foods was used in one patient with L‐dopa responsive THD experiencing fatigue and end‐dose dystonia [38]. The patient responded favorably to this dietary intervention, showing a 7‐point decrease on the Unified Dystonia Rating Scale. Despite the positive results reported in this single case, a low‐protein diet is generally not recommended for children, as normal protein intake is essential for proper growth and development. L‐dopa is generally recommended to be taken at least 30 min before or 1 h after meals, as protein‐rich foods can delay and reduce its absorption. However, it is not contraindicated to take L‐dopa with food. In fact, for patients who experience side effects such as nausea or gastrointestinal discomfort, taking L‐dopa with food may help improve tolerance.

## 13 Part IIIe: Treatment: Other Supportive Therapies

### 13.1 Deep Brain Stimulation (DBS)

DBS is a surgical intervention that involves implanting electrodes in specific brain regions and connecting them to a pulse generator. Stimulation of these electrodes can modulate abnormal patterns of neural activity. DBS targeting the internal globus pallidus and the subthalamic nucleus (STN) has been found to be effective and safe targets in advanced Parkinson's disease with medically refractory L‐dopa‐induced motor complications.

Bilateral STN DBS has been reported in a single patient with a putative diagnosis of THD [54]. Uncertainties regarding his diagnosis emerge from the case description, including the report of a single unidentified mutation in the TH gene and an atypical clinical course with progressive symptoms after an initial response to L‐dopa and development of drug‐induced dyskinesia 3 years after the initiation of treatment, suggestive of a neurodegenerative process. Of note, the finding of low levels of HVA in CSF has been observed in a number of neurological conditions and in degenerative infantile parkinsonism [55, 56].

There is no evidence available that DBS is effective for the treatment of parkinsonism in THD in case of incomplete control of symptoms or poor tolerance to treatment.

## 14 Part IIIf: Treatment: Drugs to Avoid

### 14.1 Antiemetic and Antipsychotic Drugs

Antiemetic and antipsychotic drugs acting as central dopamine antagonists are not recommended in THD, due to their potential to worsen symptoms related to dopamine deficiency. While pathophysiological considerations suggest potential side effects, there is a lack of available literature or clinical experience to guide their use.

## 15 Part IIIg: Treatment: Acute situations

### 15.1 Management of Acute Situations

Abrupt discontinuation of dopaminergic drugs should be avoided. This is to prevent the potential development of acute akinesia or dystonic crisis.

This challenge is also relevant in patients undergoing scheduled or emergency surgery. If needed, symptomatic treatment options, such as intravenous administration of benzodiazepines, can be considered.

Patients experiencing an acute condition accompanied by nausea and vomiting may encounter difficulties in tolerating oral medications, potentially leading to missed doses. In the presence of nausea and vomiting, prioritizing supportive care to prevent dehydration is essential. If medical therapy is necessary, consider low‐dose domperidone as an option given its inability to cross the blood–brain barrier, thus limiting side effects in these patients.

*R#37 (GPP)*: Supportive care for nausea and vomiting should be optimal. If anti‐nausea treatment is needed, low‐dose domperidone can be considered. It is recommended not to use metoclopramide for the treatment of nausea in THD.

*R#38 (GPP)*: Motor complications due to abrupt discontinuation of dopaminergic drugs should be followed closely and managed symptomatically.

*R#39 (GPP)*: In exceptional circumstances in which a patient cannot tolerate oral medications for a prolonged period, non‐oral formulations of dopamine agonists (transdermal rotigotine, subcutaneous apomorphine) may be contemplated as a bridging treatment.

### 15.2 Dystonic Crises and Other Acute Movement Disorders

Dystonic crisis characterized by sustained and severe muscle contractions carries potential risks including compromised airway and metabolic complications such as rhabdomyolysis, which may result in acute renal failure. Although there have been no reported cases of dystonic crisis in patients with THD, the potential for such crises exists as mentioned earlier. The management approach to dystonic crisis in THD aligns with the general approach, involving admission to an intensive care unit, supplementation of fluids and nutrition, sedation (typically with benzodiazepines), and respiratory support as necessary [57, 58].

Other acute severe movement disorders include episodes of massive disabling dyskinesia [19, 30, 47, 59] or even choreic status [40]. The management typically entails reducing the dosage of L‐dopa and, in patients on dopamine agonists, decreasing or discontinuing the dopamine agonist to better control the abnormal movements.

Akinetic status has been described in one patient as a paradoxical reaction to rotigotine treatment [40].

*R#40 (GPP)*: Dystonic crisis in THD could be a potentially life‐threatening condition and should be treated promptly.

*R#41 (GPP)*: Approach for status dystonicus is not THD specific. Follow the local guidelines. Dopaminergic treatment should be continued. Antidopaminergic drugs must be avoided.

*R#42 (GPP)*: Paradoxical responses to dopaminergic agents are managed by reducing or discontinuing the offending agent.

Recommendations on the management of drug‐induced dyskinesia have been discussed earlier.

## 16 Part IV: Follow‐Up and Long‐Term Management

### 16.1 Follow‐Up Visits

There are only a few publications available that give recommendations on topics that should be included in standardized follow‐up visits of patients with THD [31]. Therefore, only conditional recommendations or GPP based on the experience of the authors can be given.

The authors of this guideline recommend a life‐long, systematic follow‐up for all patients with THD to achieve optimal development and to prevent or avoid treatment side effects. Follow‐up visits should be done ideally in a multidisciplinary setting by a (child) neurologist with experience in movement disorders or neurometabolic diseases. They should be conducted at least yearly. A higher frequency is required in infants and young children who require frequent dose adjustments due to the initial dose titration or weight gain (e.g., infants every 3 months; older children at least every 6 months).

Follow‐up visits should assess the following aspects.

#### 16.1.1 Clinical Follow Up

- *Anthropometric data*: Length, weight, BMI, subcutaneous fat, head circumference.
- *Neurological symptoms*: Motor milestones, epileptic seizures, oculogyric crises, autonomic symptoms (sweating, fever, nausea, vomiting, stool frequency, micturition frequency, sleep, behaviour), eating habits, speech development.
- *General medical history*: Infections, narcotics or alcohol abuse, pregnancy, conception.
- *Current medication*: Regular intake, any symptoms related to overdose/underdose.
- *Vaccination status*: According to local recommendations.
- *Orthopaedic complications*: Such as contractures, luxation, scoliosis.
- *Endocrinological symptoms*: Symptoms associated with hyperprolactinemia or prolactinoma such as galactorrhoea, hypogonadism, decreased libido, impotence, and secondary amenorrhoea.
- *Neurocognitive assessment*: In addition to established age adapted tests, local protocols should be followed (e.g., follow‐up for premature born).
  Integration and inclusion in kindergarten, school, education, occupation (if applicable).
- *Psychological burdens*: Psychological support for patients and families should be offered.

*R#43 (Conditional)*: Endocrinological symptoms (e.g., galactorrhoea, hypogonadism) should be carefully assessed as part of the standard of care.

#### 16.1.2 Biochemical Follow‐Up

- *CSF*: HVA should not be used routinely to guide therapeutic decision since CSF metabolites may not correlate with the clinical response. Analysis of neurotransmitter metabolites in the CSF can be considered in case of unexpected clinical signs, inadequate response to therapy or suspected treatment non‐adherence.

*R#44 (conditional)*: Lumbar puncture should not be performed routinely, as the CSF metabolites may not correlate with the clinical response.

*R#45 (conditional)*: 5‐MTHF in CSF can be decreased due to long‐term L‐Dopa/DCI intake. 5‐MTHF should be included in every CSF analysis.

- *Plasma*: Prolactin measurement is not recommended to evaluate the response to treatment but should be considered to monitor L‐dopa refractory hyperprolactinemia (also see #R15).

#### 16.1.3 Radiological and Technical Follow‐Up

Clinical and technical examinations (EEG, MRI) and consultations (neurology, psychiatry) should be carried out in case of suspected epilepsy and/or psychiatric or psychological abnormalities, respectively.

- *Cranial MRI*: Radiological diagnostics are not recommended routinely but should be performed depending on clinical presentation.

*R#46 (conditional)*: Cranial MRI should be considered in case of the onset of new clinical symptoms or constantly elevated prolactin levels under adequate L‐dopa/DCI supplementation.

- *EEG, ECG, and/or echocardiography*: Only for the clarification and monitoring of corresponding clinical signs and symptoms.

#### 16.1.4 Long‐Term Complications

Orthopaedic complications (e.g., contractures, luxations, scoliosis) [49], infections [13, 43, 45, 60, 61], side effects of drugs [3, 16, 19, 40, 48, 62], and others like gastrointestinal problems or autonomic dysfunction [17, 39] are reported among long‐term complications. Detailed information in big cohorts with systematic follow‐up is missing.

*R#47 (research)*: For a better understanding of long‐term complications in THD (e.g., orthopaedic complications, infections, side effects of medications, gastrointestinal problems, autonomic dysfunction), documentation of follow‐up examinations in patient registries can be an important source of real‐world data for further research.

## 17 Part V: Special Situations

### 17.1 Infections

Infections and/or fever might lead to a worsening of symptoms in THD including clinical regression and an increase of L‐dopa induced dyskinesia [13, 43, 45, 60, 61].

*R#48 (strong)*: Intercurrent infections and/or fever should be carefully monitored and treated to prevent worsening of symptoms of THD.

*R#49 (GPP)*: Patients with THD should receive vaccinations according to local vaccination programs.

*R#50 (GPP)*: Educational and health care plans should be implemented in the school setting if needed.

### 17.2 Anaesthesia

From the metabolic perspective, standard protocols for anaesthesia can be followed. Drugs acting as central dopamine antagonists as well as antidopaminergic drugs should be avoided. Pre‐anaesthetic assessment is recommended, and the patient's comorbidities should be considered. Missing treatment dosages as well as stress (fever/metabolic/psychological) should be avoided as much as possible. Autonomic functions should be monitored during anaesthesia.

*R#51 (GPP)*: The patient's comorbidities should be considered for anaesthesia. After the operative procedure, standard oral treatment should be continued as soon as possible.

### 17.3 Genetic Counselling

THD is an inherited disorder. Therefore, genetic counselling should be offered to parents and/or patients according to local protocols and possibilities. If the patient wishes to have children or is pregnant, genetic consultations (periconceptional, postconceptional, during, and after the pregnancy) should be considered and offered.

*R#52 (strong)*: Genetic counselling should be offered to patients and their families.

### 17.4 Pregnancy

There are few cases published regarding the management of pregnancy in patients with THD [36, 48, 63]. During pregnancies, it is important to keep disease‐related symptoms under control. Dopaminergic treatment should continue at the lowest effective dose for adequate symptom control, and the dose adjusted if needed. A multidisciplinary team (metabolic consultant, neurologist, gynecologist, geneticist) should intensively supervise the pregnancy.

Before a pregnancy, genetic screening of the partner for THD (see genetic counselling) should be offered. No evidence of fertility issues is available in the literature. Research in this area is needed.

*R#53 (strong)*: Dopaminergic treatment should continue during pregnancy, at the lowest effective dose for adequate symptom control.

## 18 Part VI: Social Issues and Transition

### 18.1 Education

THD can lead to a global developmental delay/intellectual disability requiring special physical and educational support settings. Additionally, medication must be taken regularly. This can lead to special requirements for kindergartens and schools.

### 18.2 Transition

There is currently no literature available regarding the management of the transition from childhood to adulthood in THD. In general, the transition to adult care involves a multidisciplinary team and demands both time and staff resources. Transitioning is an ongoing process rather than a single event. Patients must receive thorough information about their disease and its progression. Important factors to consider during this period include shifts in the roles of patients, parents, and caregivers, as well as psychological and social considerations. Legal guardianship should be clarified, depending on the degree of disability [64]. The transition of patients with THD to adult care should be planned and performed in specialized centres in a multidisciplinary setting.

### 18.3 Patient Advocacy Groups

There are several non‐profit parental umbrella organizations dedicated to rare neurotransmitter diseases, including THD:

– Lil' Brave One—Hrabrisa (Serbia), www.hrabrisa.rs/en
– De neu (Spain), www.deneu.org
– Group for neurotransmitter‐related disorders (Germany), www.dig‐pku.de/wcf/index.php?neurotransmitterstoerungen‐nts
– EURORDIS, a non‐governmental patient‐driven alliance of organizations and individuals active in the field of rare diseases in Europe, https://www.eurordis.org.

Regular updates on patient advocacy groups can be found under https://intd‐online.org/patients.

In addition, there is an increasing amount of active social media accounts from affected families addressing available supportive settings and daily life aspects of children with THD, including disability and inclusion.

These associations and groups serve as vital pillars of support for families affected by rare neurotransmitter disorders. Their efforts include patient advocacy, awareness raising, organizing family meetings, and supporting scientific research. The core aim of these organizations is to provide support and to improve procedures in diagnosis, treatment, and all‐around care. It is recommended that parents and caregivers of TH deficient patients should be informed about the availability of patient advocacy groups for this rare disorder.

## 19 Conclusion

This is the first consensus guideline for the diagnosis and management of THD. All recommendations are based on the available literature and on expert opinion and were formulated in a transparent consensus process by the iNTD guidelines working group. The guideline is aimed at clinicians, metabolic biochemists, and paramedical professionals involved in the care of patients with THD. We hope it will help to harmonize clinical practice and lead to standardization and improvement of the care of patients with THD worldwide.

## Author Contributions

T.O., M.S.B., A.G.‐C., K.J., T.S.P., R.P., J.K., and O.K.H.: conception and design of the article. K.J. and M.S.B. literature review was conducted. T.O., M.S.B., A.G.‐C., R.P., T.S.P., J.K., and O.K.H. drafted the manuscript and coordinated (sub‐)group communications. T.O. and A.G.‐C. were the leaders of the guideline working group. All authors were active members of the iNTD network. All authors read, critiqued, and approved the final manuscript.

## Ethics Statement

The authors have nothing to report.

## Consent

The authors have nothing to report.

## Conflicts of Interest

The authors declare no conflicts of interest.

## Supporting information


**Figure S1:** Systematic literature search flow chart.

**Data S1:** List of key questions per working group.

## Data Availability

The data that support the findings of this study are available from the corresponding author upon reasonable request.
