# Supplementary figures and images for: Consensus Guideline for the Diagnosis and Treatment of Tyrosine Hydroxylase (TH) Deficiency
Source: J Inherit Metab Dis. 2025 Nov 10;48(6):e70106. doi: 10.1002/jimd.70106 (PMC12603479; doi:10.1002/jimd.70106)

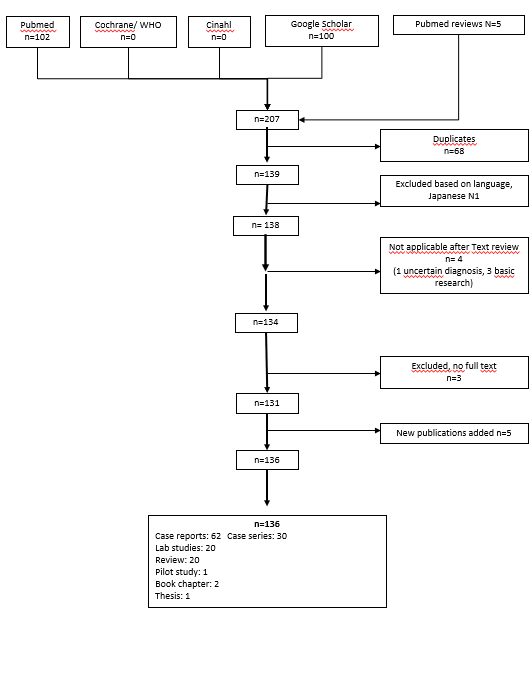

Supplement: Supplementary file 1 — Figure S1: Systematic literature search flow chart. [file JIMD-48-e70106-s002.docx]
