# Supplementary material for: Consensus Guideline for the Diagnosis and Treatment of Tyrosine Hydroxylase (TH) Deficiency
Source: J Inherit Metab Dis. 2025 Nov 10;48(6):e70106. doi: 10.1002/jimd.70106 (PMC12603479; doi:10.1002/jimd.70106)
Supplement: Supplementary file 2 — Data S1: List of key questions per working group. [file JIMD-48-e70106-s001.docx]

**Main topics:**

I Clinical presentation -1^st^ Group

IIa Diagnosis: laboratory tests - 2^nd^ Group

IIb Diagnosis: imaging and other

III Treatment -3^rd^ Group

IV Complications and long-term management -4^th^ group

V Social issues and transition -4^th^ group

VI Special situations -4^th^ group

**List of Key Questions per working group**

**Part I: Clinical Presentation (working group 1):**

1.1: **Published clinical symptoms**

1.1.1: which signs and symptoms are described in THD before treatment?

1.1.2: which signs and symptoms are described in THD during follow-up?

1.1.3: what is the age of onset of each symptom/ sign?

**Table 1: symptoms and signs in clinical presentation of THD**

| A | Neurological disorders | | |
| --- | --- | --- | --- |
|  | A.1 | Movement disorders/extrapyramidal disorders | - Hypokinesia/Bradikinesia - Rigidity - Hypotonia - Hypertonia - Dystonia - Dyskinesia - Chorea - Tremor - Complex hyperkinetic movements - Spastic paraparesis - Diurnal fluctuations of motor symptoms - …. |
|  | A.2 | Eye movement disorders | - Oculogyric crisis - Palpebral ptosis - ….. |
|  | A.3 | Speech development | - Language development |
|  | A.4 | Cognitive development | Describe |
|  | A.5 | Psychiatric and behavioural disorders | - Autism, - ADHD - OCD - Anxiety disorder - Depression - …. |
|  | A.6 | Developmental milestones | - Motor (describe) - Language ... |
|  | A.7 | Sleeping disturbances | - Hypersomnia - Insomnia - Fatigability - Diurnal fluctuations - ... |
|  | A.8 | Autonomic disorders | - Ptosis - Temperature instability - Sweating - Drooling (including hypersalivation) - Nasal congestion - …. |
|  | A.9 | Epilepsy | Describe |
|  | A.10 | Other | Include any other reported neurological symptom/ sign |
| B | Non- neurological disorders | | |
|  | B.1 | Upper-airway disorders | - Laryngomalacia - ... |
|  | B.2 | Gastro-intestinal disorders | - Constipation - Diarrhoea - Feeding problems - Tube feeding - ... |
|  | B.3 | Endocrine disorders | - Growth Hormone - Thyroid function - Hyperprolactinaemia - …. |
|  | B4 | Anthropometric disturbances | - Microcephaly - Growth retardation - ….. |
|  | B.5 | Other | Include any other reported non-neurological symptom/ sign |
| C | Pregnancy & Prenatal symptoms | | |
|  | C.1 | Pregnancy | - unusual fetal movements - prematurity - complication during delivery |
|  |  | Fetal distress |  |
|  |  |  |  |

1.2: Age at diagnosis

1.2.1 What is the age of diagnosis of reported patients with THD?

1.3. Different phenotypes according to clinical severity/gender/CSF levels

1.3.1: Are there clinically different phenotypes in THD?

1.3.1.1. severe/ moderate/ mild?

1.3.1.2. neonatal/ infantile/ childhood?

1.3.1.3. responsive/unresponsive?

1.3.1.4. Is the differentiation in THD type A or B still state of the art?

1.3.2: Are there gender-related differences in THD?

1.3.3. Is there a correlation between the values of CSF neurotransmitter metabolites and the phenotype?

1.4: Is there a genotype/phenotype correlation in THD?

**Part IIa: Diagnosis, Laboratory Tests (Working Group 2)**

2.1 Describe the following available diagnostic tests:

2.1.1 Lumbar puncture

2.1.1.1 Describe for each of the measurements in **table 2:**

a. Diagnostic value

b. Recommended method and handling

c. Centers where this test can be performed

d. Reference value

e. Medication that should be stopped before initial diagnostic test

**Table 2: CSF measurements in THD**

| 1 | Neurotransmitter metabolites (HVA/5-HIAA/5-HTP/3-OMD, other) |
| --- | --- |
| 2 | Pterines |
| 3 | Folate |
| 4 | Other (describe…) |

2.1.2 THD-activity measurement in Erythrocytes/ Fibroblasts:

a. What is possible?

b. Recommended method and handling

c. Centers where this test can be performed

d. Reference value

e. Medication that should be stopped before the initial diagnostic test

2.1.3 Genetic Diagnosis:

2.1.3.1. Gene analysis of *TH:*

a. Diagnostic value

b. Recommended method and handling

c. Centers where this test can be performed

d. List of mutations

2.1.3.2. Panel diagnostics:

a. Diagnostic value, what is known?

2.1.3.3. Whole exome sequencing

a. Diagnostic value, what is known?

2.1.4. Blood tests:

2.1.4.1. Prolactin

a. Diagnostic value

b. Reference values

Can this test be used to evaluate treatment response? (follow-up)

2.1.4.2. Monitoring for secondary deficiency states (folate deficit/methionine deficit etc…)

a. Describe

2.1.5 Urine Tests

2.1.5.1. Vanillactic acid

a. Diagnostic value

2.1.5.2. Measurement of dopamine, norepinephrine, and epinephrine in urine

a. Diagnostic value

2.1.5.3. Melatonin

a. Diagnostic value

b. Reference values

Can this test be used to evaluate treatment response? (follow-up)

2.1.5.4. Other (experimental studies /animal models…)

b. Describe

2.1.6 Other tests (e.g. saliva).

2.1.7. Is there any evidence for newborn screening tests?

2.1.8. Prenatal screening. Is there any evidence for genetic studies on prenatal level? Prenatal studies on experimental level (mouse models).

**Part IIb: Diagnosis; imaging and electroencephalography (working group 2)**

2.1.8 Radiological examinations

2.1.8.1. Magnetic resonance imaging (MRI) of the brain

a. What abnormalities are described?

b. What is the diagnostic value?

2.1.8.2. Nuclear imaging

a. What techniques are described?

b. What abnormalities are described?

c. What is diagnostic values?

2.1.9. Electroencephalography

a. What abnormalities are described?

b. What is the diagnostic value?

**Diagnosis, general:**

- What are mandatory tests for definite diagnosis of THD?

**Part III: Treatment (Working Group 3)**

Part IIIa: medical treatment of THD

3.1. Describe for all the following possible drug options for chronic/ maintenance drugs in THD **table 3:**

1. Evidence: describing effects for specific outcomes (listed in Table 1)
2. Dosage
3. Effects
4. Side Effects
5. Subset of patients for which this drug has better or worse outcome (if any)

**Table 3: list of different drugs used in maintenance therapy of THD**

| **NO** | **Class** | **Specific drugs** |
| --- | --- | --- |
| **Primary:Levodopa supplementation** | | |
| 1 | Levodopa | na |
| 2 | Levodopa + peripheral DDC inhibitor (Carbidopa or benserazide) | na |
| **Other dopaminergic therapy** | | |
| 3 | Dopamine Agonists | Bromocriptine |
|  |  | Pramipexol |
|  |  | Ropinirol |
|  |  | Rotigotine patches |
|  |  | Pergolide |
|  |  | Cabergolide |
| 4 | MAO-inhibitors | Selegiline |
|  |  | Tranylcypromine |
|  |  | Other |
| 5 | COMT-inhibitors | Describe |
| 6 | Other | Amantadine |
| **Symptomatic treatment (dystonia, mood, behaviour, attention, sleep…)** | | |
| 7 | Anti-cholinergic agents | Trihexyphenidyl |
|  |  | Biperiden |
|  |  | Others |
| 8 | Anti-epileptic drugs | Gabapentin |
| 9 | Benzodiazepines | Diazepam, lorazepam, clonazepam… |
| 10 | Alpha-adrenoreceptor agonists | Clonidine, others? |
| 11 | Selective Serotonin Reuptake Inhibitors | Fluoxetine, sertraline, paroxetine, escitalopram… |
| 12 | Stimulants | Methylphenidate… |
| **Other metabolic treatment** | | |
| 13 | Folinic acid | na |
| 14 | Melatonin | na |
|  | Others? |  |

**Part IIIb: non-medical treatment in THD**

3.2 What paramedical therapy is recommended in THD?

*If no evidence is available specific to THD, search for evidence for children with chronic neurological disorders in general, e.g. cerebral palsy)*

3.2.1. Physiotherapy

3.2.2. Speech therapy

3.2.3. Dietary

3.2.4. Occupational therapy

3.2.5 (Neuro)Psychological therapy

3.3 What is the evidence for surgical treatment options in THD?

3.3.1. Deep brain stimulation

3.3.2. Gene Therapy

3.4 Contra-indications

3.4.1. What drugs are to be avoided in THD?

3.4.1.1. Neuroleptics

3.4.1.2. Other…

3.5 What drugs can be used in acute settings, e.g. for treatment of acute/subacute dystonic deterioration?

a. Described dosage, effect, and evidence

3.5.1. Baclofen

3.5.3 Benzodiazepines

3.5.4. Chloralhydrate

3.5.5. Phenobarbitone

3.5.6. Cannabinoids

3.5.7. Clonidine

3.5.8. Other

3.6 Special situations: prolonged fasting or inability to take oral medications

**Treatment, general:**

- What is the proposed algorithm for maintenance therapy of THD?

**Part IV: Complications and long-term management of THD patients (working group 4)**

4.1. Long-term complications:

4.1.1 Describe for each of the following complications their prevalence, proposed treatment and proposed monitoring:

4.1.1.1. Orthopedics complications (e.g. contractures, luxations, scoliosis)

4.1.1.2. Infections

4.1.1.3. Related to long-term medication

4.1.1.4. Other long-term complications (e.g. gastrointestinal autonomic dysfunction)

4.2. Follow-up visits

4.2.1. Which topics should be covered on follow-up visits?

4.2.2. What monitoring tests are recommended and how should they be performed?

4.2.2.2.1. Nutritional monitoring

4.2.2.2. Orthopedic monitoring

4.2.2.3. Lab tests (if any) ->endocrinological assessment, prolactin

4.2.2.4. Specific biochemical monitoring (lumbar puncture?)

4.2.2.5. Radiological monitoring

4.2.2.6. Cognitive assessment

4.2.2.7. Other (I.e. psychiatric assessment, monitoring for seizures, imaging?)

**Long-term monitoring, general:**

- What is the proposed long-term management approach for THD?

**Part V: Social issues and transition (working group 4)**

What is known about, or can be recommend, regarding:

5.1. Psychological support for families

5.2. Genetic counseling

5.3. Parental organizations

5.3.1. Contact information

5.4. Transition to adulthood and puberty issues (follow if puberty comes naturally; are there changes related to gender)

5.5. Fertility issues

5.6 Educational needs

**Part VI: Special situations (working group 4)**

Describe what is known of, and recommended care in:

6.1. Anesthesia for interventions (Describe what is known of, and recommended care in anesthesia for interventions drugs to avoid is handled by treatment group).

6.2. Intensive care management (Describe what is known of, and recommended care in intensive care management (incl. status dystonicus with ICU admission).

6.3. Pregnancy (Describe what is known of, and recommended care in pregnancies).
